# Supplementary material for: Para-Halogenation of Amphetamine and Methcathinone Increases the Mitochondrial Toxicity in Undifferentiated and Differentiated SH-SY5Y Cells
Source: Int J Mol Sci. 2020 Apr 18;21(8):2841. doi: 10.3390/ijms21082841 (PMC7215714; doi:10.3390/ijms21082841)
Supplement: Supplementary file 1 [file ijms-21-02841-s001.pdf]

International Journal of
Molecular Sciences

MDPI

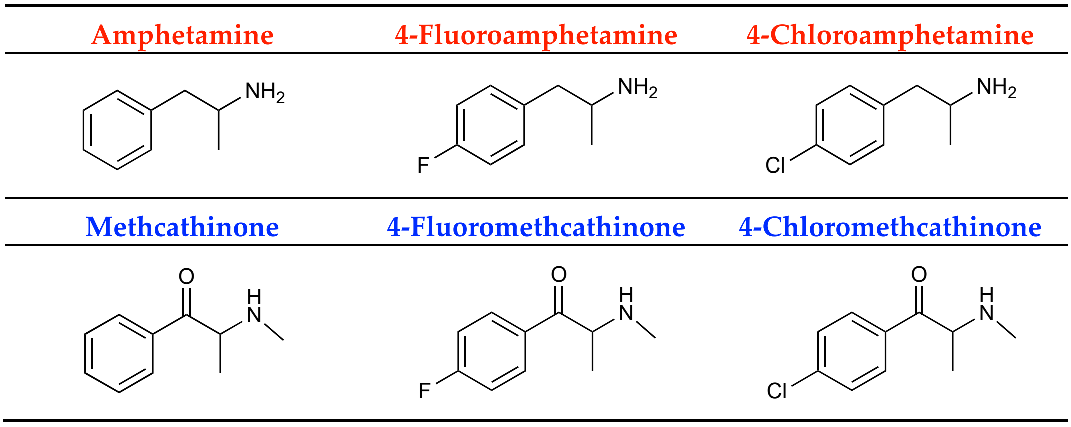

Figure S1. Chemical structures of *para*-halogenated amphetamine and methcathinone derivatives.

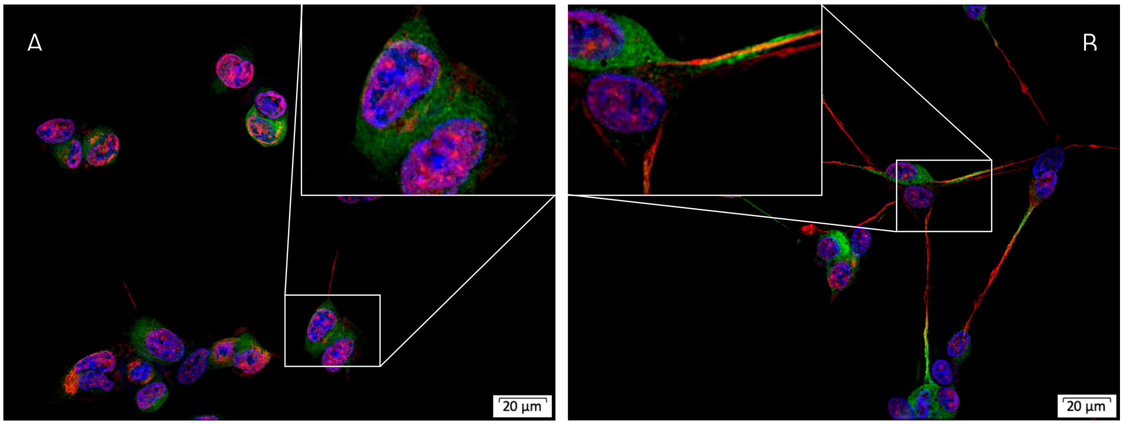

Figure S2. Differentiation of SH-SY5Y cells. Differentiation was performed by treatment with all-trans-retinoic acid (ATRA) and brain derived neurotrophic factor (BDNF). (**A**) Undifferentiated and (**B**) differentiated SH-SY5Y cells.

Table S1. Summary of the toxicity. The concentrations (mM) indicated in table correspond to the lowest concentration where a significant toxic effect was recorded in undifferentiated (und), and differentiated (diff) SH-SY5Y cells exposed to amphetamine (Amph) and methcathinone (MC) derivatives.

Drug	Amph		4-FA		PCA		MC		4-FMC		4-CMC	
Cell	und	diff	und	diff	und	diff	und	diff	und	diff	und	diff
Cytotoxicity [mM]	2		2	2	0.5	0.2					2	2
ATP depletion [mM]	2		1	2	0.2	0.5					0.5	1
MMP [mM]			1	0.5	0.5	0.2					0.5	2
OCR [mM]					0.05	0.1					0.5	0.2
O^- production [mM]			2		0.5	2					2	2
Apoptosis [mM]					0.2	0.1					2	1
